# The Transcription Factor VdHapX Controls Iron Homeostasis and Is Crucial for Virulence in the Vascular Pathogen Verticillium dahliae

**DOI:** 10.1128/mSphere.00400-18

**Published:** 2018-09-05

**Authors:** Yonglin Wang, Chenglin Deng, Longyan Tian, Dianguang Xiong, Chengming Tian, Steven J. Klosterman

**Affiliations:** a Beijing Key Laboratory for Forest Pest Control, College of Forestry, Beijing Forestry University, Beijing, China; b Agricultural Research Service, United States Department of Agriculture, Salinas, California, USA; Carnegie Mellon University

**Keywords:** HapX, *Verticillium dahliae*, fungal virulence, iron homeostasis, vascular wilt

## Abstract

Iron homeostasis is essential for full virulence and viability in many pathogenic fungi. Here, we showed that the bZip transcription factor VdHapX functions as a key regulator of iron homeostasis for adaptation to iron-depleted and iron-excess conditions and is required for full virulence in the vascular wilt fungus, Verticillium dahliae. Deletion of *VdHapX* impaired mycelial growth and conidiation under both iron starvation and iron sufficiency. Furthermore, disruption of *VdHapX* led to decreased formation of the long-lived survival structures of V. dahliae, known as microsclerotia. Expression of genes involved in iron utilization pathways and siderophore biosynthesis was misregulated in the Δ*VdHapX* strain under the iron-depleted condition. Additionally, the Δ*VdHapX* strain exhibited increased sensitivity to high iron concentrations and H_2_O_2_, indicating that VdHapX also contributes to iron or H_2_O_2_ detoxification. The Δ*VdHapX* strain showed a strong reduction in virulence on smoke tree seedlings (Cotinus coggygria) and was delayed in its ability to penetrate plant epidermal tissue.

**IMPORTANCE** This study demonstrated that VdHapX is a conserved protein that mediates adaptation to iron starvation and excesses, affects microsclerotium formation, and is crucial for virulence of V. dahliae.

## Abstract (precis)

This study demonstrated that VdHapX is a conserved protein that mediates adaptation to iron starvation and excesses, affects microsclerotium formation, and is crucial for virulence of V. dahliae.

## INTRODUCTION

Iron is an essential trace element for almost all organisms. As an indispensable cofactor of protein function, redox-active iron participates in multiple cellular processes, such as oxidative phosphorylation and detoxification of oxidative stress (1). However, iron excess is toxic to the cell (2). Thus, iron homeostasis is finely controlled to maintain the subtle balance between uptake, storage, and utilization of iron.

Iron homeostasis is primarily maintained by two transcription factors in filamentous fungi, including the bZip transcription factor, HapX, and the GATA zinc finger transcription factor, SreA, which are interconnected by a negative transcriptional feedback loop, i.e., in Aspergillus nidulans (3, 4) and Aspergillus fumigatus (5). During iron starvation, HapX represses SreA-mediated iron utilization pathways and activates siderophore biosynthesis for iron uptake (5–7). In periods of iron sufficiency, SreA represses HapX expression and siderophore-mediated iron acquisition (3, 7). Furthermore, HapX functions by physical interaction with the heterotrimeric CCAAT-binding complex (4). HapX is also critical for iron detoxification by triggering expression of the vacuolar iron importer CccA under conditions of excess iron (7, 8). In addition to the core role of HapX in iron homeostasis, deletion of HapX leads to attenuated virulence in A. fumigatus (5), Candida albicans (9, 10), and Cryptococcus neoformans (11). In contrast, HapX of the human-pathogenic dermatophyte Arthroderma benhamiae is not a virulence determinant (12). Overall, the roles of HapX orthologs in plant vascular wilt fungi have not been well investigated thus far, except in the soilborne fungus Fusarium oxysporum (6).

Vascular wilt caused by Verticillium dahliae is a destructive plant disease that poses a threat to crop production and forest health worldwide (13, 14). The fungus infects more than 200 plant species, and an increasing number of new hosts are continually identified (15). In China, apart from the cultivated crop plants, ornamental and landscape plants like smoke trees (Cotinus coggygria) are also infested by V. dahliae (13). The fungus infects its host through the root and colonizes and propagates in xylem vessels (16). Once the plant is infected, no available fungicides can effectively treat the disease (14).

The V. dahliae life cycle comprises three stages, including parasitic and saprophytic stages in xylem and a dormant stage in the soil as long-lived survival structures known as microsclerotia, which play crucial roles in disease spread (14). The xylem transports water and soluble mineral nutrients from the roots throughout the plant. As such, xylem sap is not rich in nutrients and contains lots of organic acids, including a small amount of amino acids (17). During parasitic and saprophytic stages, as anticipated for similar plant pathogens, V. dahliae must employ its iron uptake system to compete for host iron resources.

The relatively recent availability of the V. dahliae genome sequence has greatly accelerated investigations into the mechanisms fundamental to its life cycle and disease progression (18). Multiple genes have been studied for their roles in nutrient uptake and adaptation to adverse circumstances, including iron limitation or excess. For example, FreB, the gene for which encodes a ferric reductase and which is involved in the reduction of the ferric iron to available ferrous iron, has been studied in V. dahliae (19). VdSNF1, the sucrose-nonfermenting protein kinase, was verified as a regulator of catabolic repression and the expression of genes involved in cell wall degradation in V. dahliae (20). Cpc1, a regulator of cross-pathway control, controls amino acid biosynthesis in Verticillium longisporum (21). However, it is unknown how V. dahliae copes with iron limitation and toxicity and whether the fungus coordinates iron homeostasis in response to changing iron levels via conserved iron-regulatory systems found in other fungi. The role of *VdHapX* in iron adaptation during iron starvation and sufficiency has not been elucidated in V. dahliae.

In this study, we showed that the VdHapX transcription factor is a major regulator of iron homeostasis, allowing adaptation to both iron-depleted and iron-excess conditions. Further, we revealed that VdHapX is crucial for virulence and detoxification to iron or H_2_O_2_ excess and affects microsclerotium formation. These results demonstrated a key role of VdHapX in iron homeostasis, virulence, and H_2_O_2_ detoxification in V. dahliae.

## RESULTS

### Loss of *VdHapX* decreases growth and sporulation.

A BLASTP (22) search using the amino acid sequence of HapX of F. oxysporum revealed a single significant match, VDAG_05022, in a reference genome of V. dahliae (23). This match showed significant similarity to HapX of F. oxysporum (63.1% identity) and A. nidulans (56.5% identity). *VDAG_05022* encodes a 456-amino-acid protein having a basic-leucine zipper (bZip) domain. Further phylogenetic analysis showed that VDAG_05022 is also highly homologous to other fungal HapX proteins (see Fig. S1 in the supplemental material). Thus, VDAG_05022 was designated VdHapX.

10.1128/mSphere.00400-18.3 FIG S1 Phylogenetic analysis of VdHapX from Verticillium dahliae and related orthologs. The phylogenetic relationship between the VdHapX orthologs from various fungi was constructed by neighbor joining with 1,000 bootstrap replicates, using MEGA version 6.0 (32). The sequences used in the analyses were all obtained from the NCBI database. Download FIG S1, file, MB. Copyright © 2018 Wang et al. 2018 Wang et al. This content is distributed under the terms of the Creative Commons Attribution 4.0 International license.

**FIG 1:**
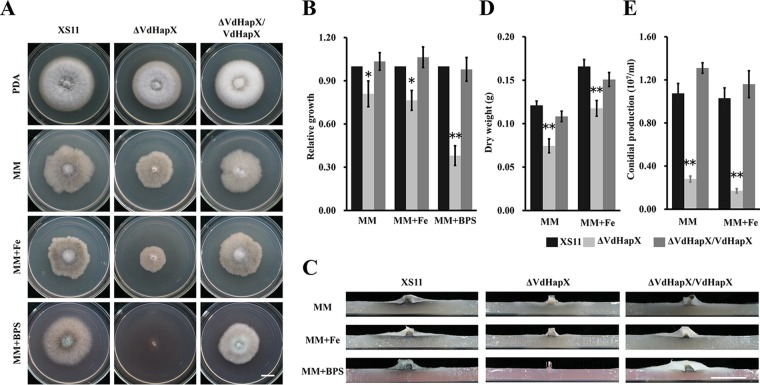
Deletion of *VdHpaX* impairs hyphal growth and conidiation in Verticillium dahliae. (A) Colonies of the wild-type strain (XS11), the Δ*VdHapX* strain, and the complemented strain (Δ*VdHapX*/*VdHapX*) grown on potato dextrose agar (PDA) and minimal medium (MM) plates for 14 days at 25°C. The indicated strains were incubated on MM plates with 0.06 mM Fe^3+^ and 0.4 mM BPS, respectively. (B) Relative mycelial growth of the indicated strains on MM with or without iron. The data were obtained by measuring the diameter of fungal colonies and were normalized to the growth of XS11 on MM. (C) Vertical dissection of the colonies on MM plates in the presence or absence of iron. (D) Relative dry biomass of the respective strains grown in liquid MM with or without iron for 14 days at 25°C. (E) Conidial production of the strains after growth for 7 days on liquid MM with or without iron. The error bars represent standard deviations based on three independent replicates, and asterisks represent signiﬁcant differences (*P* < 0.01). Bar, 1 cm.

To study the role of VdHapX in V. dahliae, two *VdHapX* mutant strains (designated Δ*VdHapX*-11 and Δ*VdHapX*-12) were identified, and deletion of *VdHapX* was confirmed in both (Fig. S2). In addition, the wild-type *VdHapX* copy was ectopically reintroduced into the Δ*VdHapX* strain, yielding the Δ*VdHapX*/*VdHapX* complemented strain (Fig. S2). The Δ*VdHapX* strain displayed no obvious growth defect on potato dextrose agar (PDA), but the mycelial growth was reduced even under the iron-replete condition (Fig. 1A). Aerial growth was eliminated in the presence of the iron chelator bathophenanthroline disulfonate (BPS) (Fig. 1A and C). Furthermore, the Δ*VdHapX* strain showed strikingly reduced radial growth in the presence of elevated iron levels (0.06 mM) or BPS (0.4 mM) compared with the growth of wild-type strain XS11 (Fig. 1B). In iron-starved or iron-replete liquid culture, the Δ*VdHapX* strain significantly decreased the biomass production to 61% or 70% of XS11, respectively (Fig. 1D). Analysis of conidiation revealed obviously reduced conidiation of the Δ*VdHapX* strain during iron starvation or sufficiency. Deletion of *VdHapX* resulted in the production of only 30% of the amount of conidia, compared with XS11 (Fig. 1E). In addition, the Δ*VdHapX*/*VdHapX* complemented strain restored the respective phenotypes similar to those of XS11 in all experiments (Fig. 1). These results demonstrated that VdHapX is required for appropriate growth and conidiation under iron-starvation conditions.

10.1128/mSphere.00400-18.4 FIG S2 Confirmation of the Δ*VdHapX* deletion mutant in Verticillium dahliae. (A) Screening of the VdHapX gene deletion mutant by PCR. Using the internal screening primer pair PL954/PL955, a 154-bp product was amplified from only the genomic DNA extracted from the wild-type strain XS11 and the complemented strains of VdHapX, but no such product was observed when PCR was performed on DNA extracted from the Δ*VdHapX*-11 and Δ*VdHapX*-12 strains. (B) The internal screening primer pair PL954/PL955 confirmed *VdHapX* replacement using reverse transcription-PCR. Primer pair VdBt-up/VdBt-down was used to verify the quality of the genomic DNA and the cDNA of all strains in this assay. (C) Confirmation of *VdHapX* deletion mutants by Southern blotting. Genomic DNA of XS1, Δ*VdHapX*-11, and Δ*VdHapX*-12 was digested with KpnI digestion. Finally, a 3.1 kb band in the wild-type strain and a 1.2 kb band in the deletion mutants Δ*VdHapX-11* and Δ*VdHapX-12* are shown. Download FIG S2, TIF file, 1.27 MB. Copyright © 2018 Wang et al. 2018 Wang et al. This content is distributed under the terms of the Creative Commons Attribution 4.0 International license.

### VdHapX is required for transcriptional regulation of SrbA and SreA.

**FIG 2:**
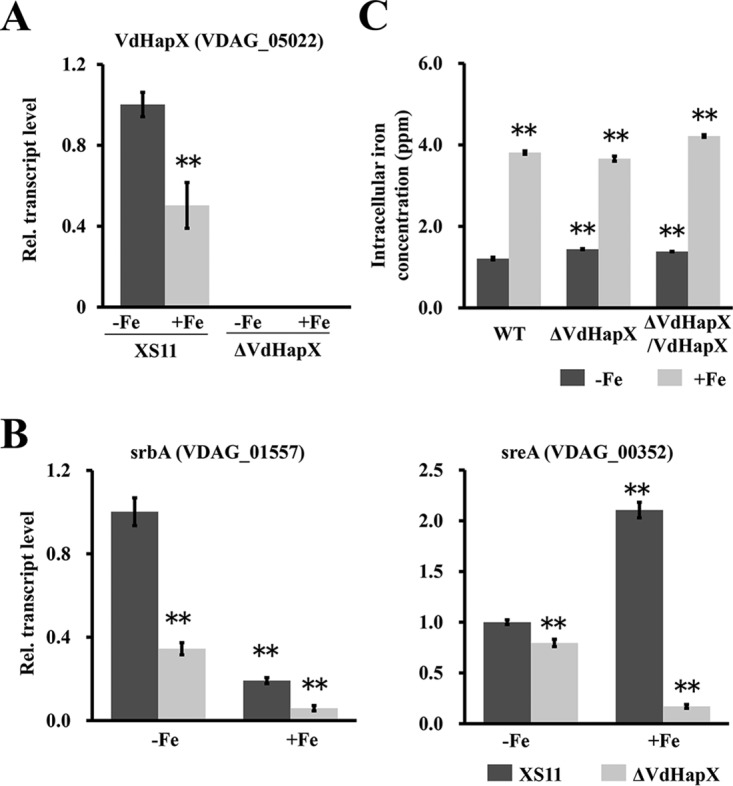
Disruption of *VdHapX* causes downregulation of iron-regulatory genes but not intracellular iron concentration in Verticillium dahliae. (A) Reverse transcription-quantitative PCR was utilized to determine transcript levels of *VdHapX* that were analyzed in XS11 and compared with those of the Δ*VdHapX* strain under iron-depleted MM (-Fe) or iron-replete MM (+Fe) conditions. The expression was normalized against the expression of the V. dahliae β-tubulin gene. Error bars indicate standard deviations from three independent experiments. (B) The expression of *srbA* and *sreA* was analyzed in XS11 and Δ*VdHapX* strains after 3 days in iron-depleted MM and transfer for 45 min to iron-depleted MM (-Fe) or iron-replete MM (+Fe). Both *srbA* expression and *sreA* expression were normalized against the V. dahliae β-tubulin gene. Values are the averages from four biological replicates, consisting of three technical replicates each. Error bars represent standard deviations. (C) Total intracellular iron concentration measurment. XS11, Δ*VdHapX* mutant, and Δ*VdHapX*/*VdHapX* complemented strains were grown in iron-depleted MM for 3 days and transferred for 45 min to iron-depleted MM (-Fe) or iron-replete MM (+Fe). The intracellular iron concentration was determined using an optical emission spectrometer. Error bars represent the standard deviations based on three independent replicates with three technical replicates. Asterisks represent signiﬁcant differences (*P* < 0.01).

We sought to investigate the role of VdHapX in the presence or absence of iron by reverse transcription-quantitative PCR (RT-qPCR). Transcript levels of *VdHapX* were signiﬁcantly upregulated (2-fold changes) in XS11 during iron starvation, indicating that *VdHapX* is an iron-repressed gene (Fig. 2A). We next examined the role of VdHapX in the transcriptional regulation of known iron-regulatory genes, such as *srbA* (VDAG_01557, sterol regulatory element binding protein) and *sreA* (VDAG_00352, GATA transcription factor). After normalization against the β-tubulin gene, Fig. 2B shows that the *srbA* transcript levels were significantly downregulated in the Δ*VdHapX* strain compared with those of XS11 under both iron starvation and iron sufficiency but were not affected by iron availability (Fig. 2B), indicating that the induction of *srbA* is independent of VdHapX. However, expression of *sreA* was highly upregulated (about 13-fold) during a 45-min shift from iron starvation to iron sufficiency in XS11. Additionally, we found that the *sreA* transcript levels were significantly upregulated under iron starvation compared with iron sufficiency by VdHapX deficiency (Fig. 2B), suggesting that VdHapX represses SreA under iron-limiting conditions. Further examination revealed that the level of intracellular iron concentration in the Δ*VdHapX* strain was comparable to that of XS11 (Fig. 2C), suggesting that VdHapX is not required for iron uptake in V. dahliae.

### Deletion of *VdHapX* leads to misregulation of siderophore biosynthesis.

**FIG 3:**
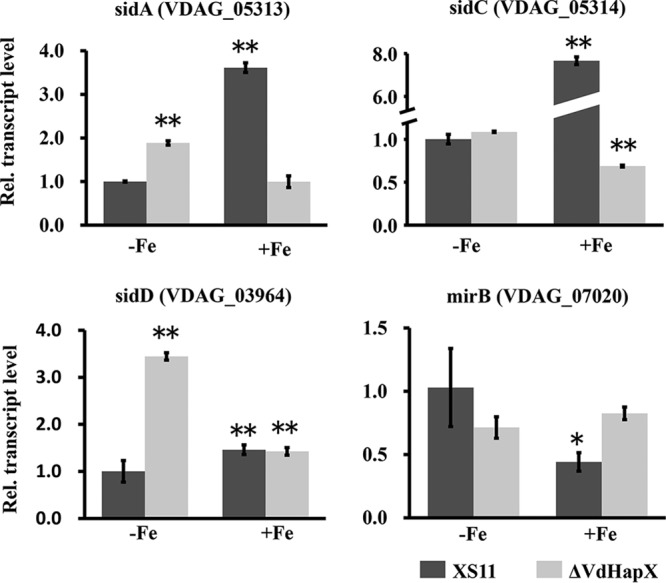
Genes involved in siderophore biosynthesis in Verticillium dahliae are misregulated in the Δ*VdHapX* strain during iron starvation. Reverse transcription-quantitative PCR analysis of postulated siderophore ferricrocin and ferrichrome C biosynthesis pathway genes based on information available in *Aspergillus* spp. (24). Transcript levels of *sidA* (*VDAG_05313*), *sidC* (*VDAG_05314*), *sidD* (*VDAG_03964*), and *mirB* (*VDAG_07020*) of V. dahliae wild-type XS11 and Δ*VdHapX* strains under iron starvation (-Fe) and iron-replete (+Fe) conditions. Data represent the means from three biological replicates. Averages of gene expression values were normalized against the β-tubulin gene. Error bars represent standard deviations. Asterisks represent signiﬁcant differences: *, *P* < 0.05; **, *P* < 0.01.

To further explore the function of VdHapX in the regulation of siderophore biosynthesis, we monitored the expression of key genes involved in iron uptake. First, we identified putative orthologs of key siderophore biosynthetic genes characterized in *Aspergillus* except *sidG* (coenzyme A [CoA]-*N*_2_-transacetylase) (24). Four homologs of these genes exist in V. dahliae, including the monooxygenase-encoding *sidA* (*VDAG_05313*), siderophore nonribosomal peptide synthetases *sidC* (*VDAG_05314*) and *sidD* (*VDAG_03964*), and the putative siderophore transporter *mirB* (*VDAG_07020*). Interestingly, the orthologs of *sidA* and *sidC* are clustered together within 21.5 kb in the genomic sequence of V. dahliae (strain VdLs.17), and the intergenic region between the two genes was 4.4 kb. The RT-qPCR analysis showed that the transcript levels of the *sidA*, *sidC*, and *sidD* orthologs were highly upregulated under iron sufficiency compared with those under conditions of iron starvation in XS11. Expression of *mirB* was highly upregulated under iron starvation compared with the levels observed during iron sufficiency in XS11 (Fig. 3). Also, the transcript levels of the *sidA* and *sidD* orthologs were highly increased in the Δ*VdHapX* strain in comparison to XS11 during iron starvation (Fig. 3); however, expression of *mirB* was decreased in the Δ*VdHapX* strain, and no differences in transcript levels of *sidC* were observed between the Δ*VdHapX* strain and XS11 under iron starvation. In contrast, the transcript level of *mirB* was slightly upregulated (about 2-fold) in XS11 during iron starvation. These results indicated that VdHapX might regulate siderophore production in a way unique to V. dahliae.

### VdHapX is involved in iron detoxification.

**FIG 4:**
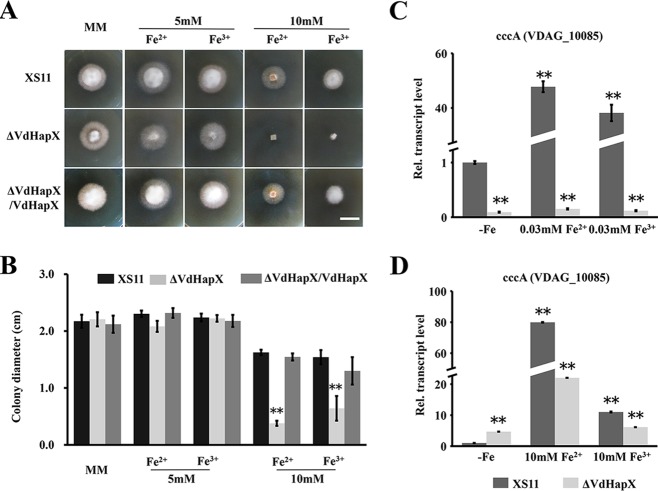
The Δ*VdHapX* strain of Verticillium dahliae displays increased sensitivity to iron toxicity. (A) The wild-type XS11, Δ*VdHapX*, and Δ*VdHapX*/*VdHapX* complemented strains were grown on solid MM with specified iron availability (Fe^2+^ and Fe^3+^) conditions for 7 days at 25°C. Images of colony morphology were taken at 7 days. (B) Mycelial growth of the indicated strains grown as described for panel A. Data are representative of mean diameters of different colonies. Error bars represent the standard deviations based on three independent replicates with three technical replicates. (C and D) Expression of *cccA* encoding vacuolar iron transporter in XS11 and Δ*VdHapX* strains following normal iron conditions (0.03 mM Fe^2+^ or Fe^3+^) and high-iron conditions (10 mM Fe^2+^ or Fe^3+^) for 45 min. Averages of the gene expression values were normalized against the V. dahliae β-tubulin gene. Error bars represent standard deviations. The asterisks indicate a signiﬁcant difference at *P* < 0.01.

We next examined potential functional roles of VdHapX in the presence of excess iron. Disruption of *VdHapX* rendered V. dahliae more susceptible to iron toxicity than XS11 on high-iron medium. Notably, in the presence of 10 mM Fe^2+^ or Fe^3+^, the Δ*VdHapX* strain displayed a strong growth defect compared with XS11 and the complemented strains (Fig. 4A). Mycelial growth of the Δ*VdHapX* strain was reduced 3-fold relative to that of XS11 grown on high-iron medium (Fig. 4B). We performed RT-qPCR analyses of a *cccA* ortholog, which encodes a vacuolar iron importer (8), and found that expression of this ortholog (*VDAG_10085*) was highly induced (>300-fold change) under normal iron conditions (0.03 mM Fe^2+^ or Fe^3+^) in XS11 (Fig. 4C). However, the induction of the *cccA* ortholog was reduced under high-iron conditions (10 mM Fe^2+^ or Fe^3+^) in which the Δ*VdHapX* strain was used as background (Fig. 4D). These data indicated that VdHapX is required for the activity of iron detoxification, mainly via CccA-mediated iron storage.

**FIG 5:**
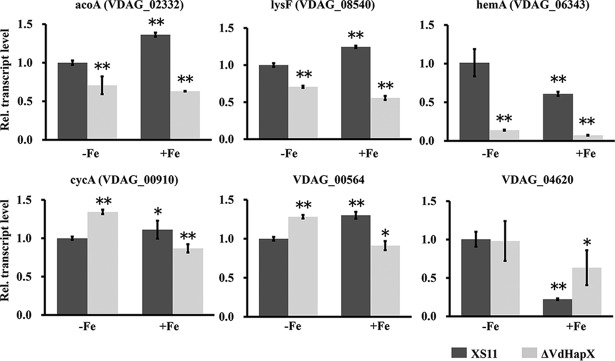
Deletion of Verticillium dahliae *VdHapX* causes deregulation of genes involved in iron use. RT-qPCR analysis was performed in XS11 and Δ*VdHapX* strains grown in liquid MM for 3 days at 25°C and transferred for 45 min to iron-depleted MM (-Fe) or iron-replete MM (0.03 mM, +Fe). Transcript levels of *acoA*, *lysF*, *hemA*, *cycA*, *VDAG_00564*, and *VDAG_04620* were analyzed under the different iron conditions shown. Averages of the gene expression values were normalized against the V. dahliae β-tubulin gene. Error bars represent standard deviations. Asterisks represent signiﬁcant differences: *, *P* < 0.05; **, *P* < 0.01.

Apart from *cccA*, previous reports showed that HapX regulates many genes involved in iron utilization in F. oxysporum (6). We inspected the V. dahliae genome using BLASTP and identified six representative genes related to iron utilization pathways in fungi. These genes included *cycA* (*VDAG_00910*), encoding cytochrome *c*; *acoA* (*VDAG_02332*), encoding aconitase; *lysF* (*VDAG_08540*), encoding homoaconitase; *hemA* (*VDAG_06343*), encoding aminolevulinic acid synthase; *VDAG_00564*, encoding isopropylmalate dehydratase; and *VDAG_04620*, encoding dihydroxy acid dehydratase. After a 45-min shift from iron-limiting to iron-replete conditions, deletion of *VdHapX* impairs the transcriptional activation of *VDAG_02332*, *VDAG_08540*, *VDAG_06343*, and *VDAG_04620*. However, the transcript levels of *VDAG_00910* and *VDAG_00564* were significantly increased in the Δ*VdHapX* strain during iron starvation, but not under iron-replete conditions, in comparison to XS11 (Fig. 5). Together, these results indicate that VdHapX contributes to detoxification of iron excesses via general upregulation of conserved iron-dependent proteins and processes.

### VdHapX inactivation increases H_2_O_2_ sensitivity.

**FIG 6:**
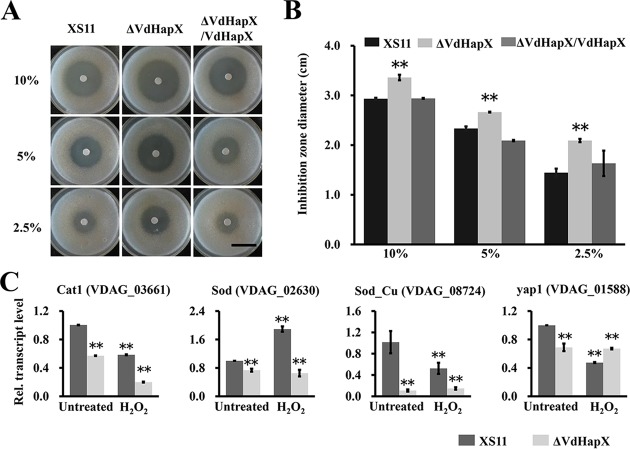
Deletion of *VdHapX* renders Verticillium dahliae more susceptible to H_2_O_2_. (A) Conidial suspensions (1 × 10^5^ spores) of each of the wild-type XS11, Δ*VdHapX*, and Δ*VdHapX*/*VdHapX* complemented strains were spread on potato dextrose agar plates. Sterile ﬁlter paper disks (5-mm diameter) were placed in the center of the plates, and 10 μl of an H_2_O_2_ solution (2.5%, 5%, and 10%) was added to each paper disk. The plates were incubated at 25°C for 4 days, and the inhibition zones were observed. (B) Bar chart of the inhibition zone of the above-described plates. Error bars represent the standard deviations based on three independent replicates. Asterisks indicate signiﬁcant differences at *P* < 0.01. (C) Expression of genes involved in H_2_O_2_ detoxification in V. dahliae, such as *cat1*, *sod*, *sod_Cu*, and *yap1*. Strains were grown in minimal medium for 3 days and then transferred for 45 min to 1 mM H_2_O_2_. Averages of the gene expression values were normalized against the V. dahliae β-tubulin gene. Error bars represent standard deviations. Asterisks represent signiﬁcant differences (*P* < 0.01).

Due to an important role of iron in detoxification of oxidative stress, we assessed whether deletion of *VdHapX* affected H_2_O_2_ sensitivity. Using H_2_O_2_ diffusion tests, we showed that the Δ*VdHapX* strain exhibited modestly increased sensitivity to different concentrations of H_2_O_2_ (Fig. 6A). Inhibition zone was determined based on an H_2_O_2_ diffusion zone diameter at 4 days postinoculation (dpi). Using a *t* test, the diﬀerence was considered signiﬁcant at *P* < 0.01 (Fig. 6B). To elucidate the molecular underpinnings of H_2_O_2_ sensitivity in the Δ*VdHapX* strain, we examined the expression levels of four orthologs of genes (*cat1*, *VDAG_03661*; *sod*, *VDAG_02630*; *sod_Cu*, *VDAG_08724*; and *yap1*, *VDAG_01588*) associated with H_2_O_2_ detoxification. With the exception of *yap1* (*VDAG_01588*), transcripts of the other three genes in the analyses were significantly downregulated in the Δ*VdHapX* strain compared to those in XS11 (Fig. 6C). These data suggested that *VdHapX* relieves oxidative stress by regulating expression of genes that detoxify H_2_O_2_.

### VdHapX positively regulates microsclerotium formation.

**FIG 7:**
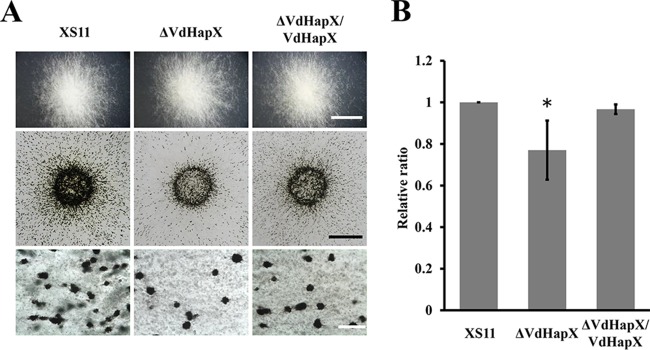
Deletion of *VdHapX* affects melanized microsclerotium formation in Verticillium dahliae. (A) Colony morphology and microscopic examination of melanized microsclerotium formation of the XS11, Δ*VdHapX*, and Δ*VdHapX*/*VdHapX* complemented strains on a BM slide at 25°C for 4 days. Bars in the top, middle, and bottom panels represent 0.5 cm, 0.25 cm, and 20 mm, respectively. (B) Bar chart showing the relative ratio of microsclerotia produced by the indicated strains. Microsclerotia formed on slides coated with BM during a 7-day incubation period. Photographs of the microsclerotia in a field were taken and converted into an 8-bit grayscale image by using Image J. Using pixels as units in the measurement area, the pixel values of the covered area were measured and the relative ratio was calculated. There were three independent fields in which microsclerotia were enumerated. Error bars represent the standard deviations based on three independent replicates. The asterisk indicates a signiﬁcant difference at *P* < 0.05.

To examine whether the Δ*VdHapX* strain was defective in microsclerotium production, we observed microsclerotium formation on solid medium. Four days after inoculation, the Δ*VdHapX* strain produced melanized microsclerotia similarly to XS11 and the Δ*VdHapX*/*VdHapX* strain (Fig. 7A). Furthermore, microscopic examination revealed that the Δ*VdHapX* strain produced 75% fewer microsclerotia than XS11 (Fig. 7B). The result indicated that VdHapX positively regulates microsclerotium formation.

### VdHapX is crucial for virulence.

**FIG 8:**
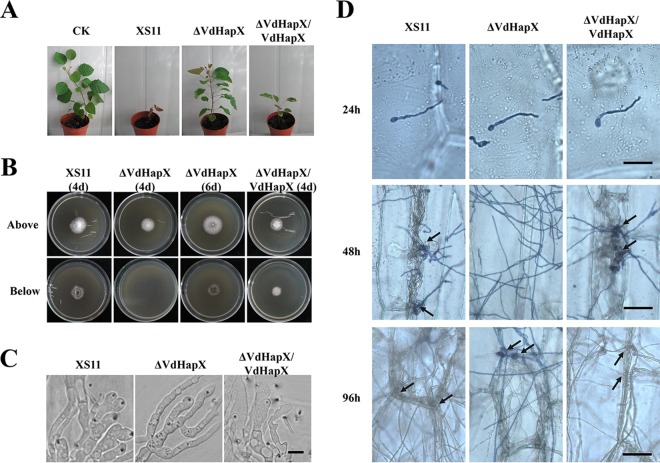
Deletion of *VdHapX* compromises full virulence on host smoke trees and penetration into plant epidermal tissue. (A) One-year-old smoke tree seedlings were inoculated and incubated for 10 min with a 10^6^-conidium/ml suspension of XS11, the Δ*VdHapX* strain, and the Δ*VdHapX*/*VdHapX* strain. The smoke tree seedlings were inoculated with distilled water (CK), also for a 10-min incubation period. All seedlings were replanted in soil for 35 days of growth, and the pictures were captured at 35 days after inoculation. (B) Colonies of each of the XS11, Δ*VdHapX*, and Δ*VdHapX*/*VdHapX* strains were grown on minimal medium overlaid with a cellophane membrane and incubated 7 dpi (top) and at 3 days after removal of the cellophane membrane (bottom). Bar, 1 cm. (C) Hyphopodia and penetration pegs formed on a cellophane membrane at 4 dpi. Bar, 10 µm. (D) Infection assays of onion epidermis examined at 24, 48, and 96 h postinoculation. Fungal hyphae were stained with trypan blue solution. Arrows indicate invasive hyphae (IH). Bars, 40 µm.

We also sought to determine whether VdHapX contributes to virulence. Compared to XS11 and the Δ*VdHapX*/*VdHapX* strain, loss of *VdHapX* severely attenuated fungal pathogenicity on smoke trees. Smoke tree seedlings inoculated by the Δ*VdHapX* strain exhibited slight chlorosis at 35 dpi, whereas the seedlings inoculated with XS11 and the complementation strain showed obvious wilt symptoms and reduced plant height (Fig. 8A). We further examined the infection process in more detail, revealing that the Δ*VdHapX* mutant exhibited delayed hyphal penetration of a cellophane membrane compared to penetration observed in XS11 and the Δ*VdHapX*/*VdHapX* strain (Fig. 8B). However, the Δ*VdHapX* strain can form hyphopodia for penetration peg development like those of XS11 and the Δ*VdHapX* complemented strain (Fig. 8C). A penetration assay on onion epidermis also revealed that deletion of *VdHapX* delayed formation of invasive hyphae (IH). Compared with XS11 and the complementation strain, IH were not observed on the onion epidermis inoculated with the Δ*VdHapX* strain at 48 h postinoculation (hpi). However, at 96 hpi, the Δ*VdHapX* strain could form IH, like those of XS11 and the complemented strain (Fig. 8D). Furthermore, we assayed whether the mutant was able to reach and proliferate within the vascular vessels, and thus, surface-sterilized stem sections were collected at 35 dpi and placed onto PDA. Although the Δ*VdHapX* strain could be reisolated from stem tissues like XS11 and the complementation strain, the rate of isolation was significantly lower than in XS11 and the complemented Δ*VdHapX* strain (data not shown). Together, these data demonstrated that *VdHapX* regulates penetration peg formation and proliferation during the initial colonization of cotton roots.

## DISCUSSION

To overcome iron deficiency or excess, pathogenic fungi have evolved sophisticated regulatory mechanisms that mediate resistance and adaptation to iron limitations or excesses. As shown here, the bZip transcription factor VdHapX is required for ion homeostasis under iron-limiting or iron-excess conditions in V. dahliae. Loss of VdHapX affects hyphal growth, microsclerotium formation, conidiation, and adaptation to oxidative stress and virulence and is necessary for adaptation to oxidative stress. Therefore, VdHapX is critically important in the life cycle and for the survival of V. dahliae.

In previous studies, HapX deficiency has resulted in significant reductions in mycelial growth on medium under an iron-limited condition, i.e., in F. oxysporum (6), A. fumigatus (5), C. neoformans (11), Arthroderma benhamiae (12), and A. nidulans (4). Compared with transcript levels observed under conditions of iron sufficiency, *HapX* and its orthologs are transcriptionally upregulated and repress iron-dependent pathways during iron starvation in fungi (4–6, 11, 12, 25). In agreement with previous studies, strains lacking *VdHapX* showed a striking reduction in growth on medium with limited iron availability. Importantly, HapX is essential for iron detoxification as well. Consequently, deletion of HapX impaired mycelial growth in V. dahliae and A. fumigatus under conditions of excess iron availability (7), demonstrating that the role of HapX in iron detoxification is conserved. Furthermore, HapX mainly activates the vacuolar iron importer *cccA* to adapt to high iron, and certain domains of HapX are necessary for the Janus-type transcription factor functions during iron starvation or under high-iron conditions as an activator or repressor (7). Similarly, in our study, knockout of *HapX* failed to result in accelerated expression of *cccA*, whose expression was highly upregulated during a shift from iron starvation to iron-rich medium in the wild type. As shown in A. nidulans, HapX functions via binding to a CCAAT motif to target promoter regions (4). Interestingly, the motif is also found in the putative promoter regions of genes regulated by VdHapX in V. dahliae (see Fig. S3 in the supplemental material), suggesting that the HapX binding motif is evolutionarily conserved. The induced expression of *cccA* when cultured under adequate iron levels was higher (>5-fold) than that observed under excess iron conditions in the wild type (Fig. 4), indicating that HapX represses or activates *cccA* depending on the ambient iron availability.

10.1128/mSphere.00400-18.5 FIG S3 Identification of the DNA motif CCAAT in the putative promoters of genes regulated by VdHapX in Verticillium dahliae. Relative positions of sequence motifs in the 2,000-bp regions upstream of the start codon of the indicated genes. Download FIG S3, TIF file, 2.18 MB. Copyright © 2018 Wang et al. 2018 Wang et al. This content is distributed under the terms of the Creative Commons Attribution 4.0 International license.

Deletion of *HapX* in V. dahliae led to activation of the siderophore biosynthesis genes *sidA* and *sidD* during iron starvation. Similarly, inactivation of HapX in F. oxysporum led to increased transcript levels of several siderophore genes during iron starvation (6). In contrast to this observation, knockout of *HapX* in V. dahliae resulted in repression of *sidC* and *mirB* under iron-limited conditions. However, there is an additional report of a HapX mutant that exhibited downregulation of genes involved in siderophore biosynthesis under iron-limiting conditions in A. fumigatus (5). As previously shown, transcription factors HapX and SreA are interconnected through a negative-feedback-loop-orchestrated transcriptional regulation of iron homeostasis and siderophore biosynthesis in fungi, such as in A. nidulans (4) and in A. benhamiae (12). Strikingly, the transcript levels of *srbA* did not vary between iron starvation and sufficiency, whereas *sreA* was primarily induced by iron sufficiency as opposed to iron starvation in the wild-type strain. Expression of *sreA* and *srbA* was markedly reduced in the Δ*VdHapX* strain compared with the wild-type strain (Fig. 2). Both SreA and SrbA have been demonstrated to contribute to the activation of siderophore production (26). However, the roles of SreA, SrbA, and HapX in siderophore production of V. dahliae require further characterization, in addition to those of the ferric reductase FreB (19). Interestingly, intracellular iron concentration is not changed in the Δ*VdHapX* strain compared with the wild-type strain. Potentially, the compartmentalization of iron could be changed in response to increases in external concentrations, though not the total iron concentration. If increased external stimulus (iron) is perceived, signaling from this stimulus may be channeled to trigger decreases in growth processes and not necessarily a simultaneous change in intracellular iron concentration. Nevertheless, data presented in this study indicate a clear role of the V. dahliae HapX in iron homeostasis and regulation of siderophore biosynthesis.

In addition to altered expression of iron-regulatory genes in the Δ*VdHapX* strain, *VdHapX* deletion in V. dahliae resulted in decreased growth and conidiation, as well as decreased melanized microsclerotium formation under both iron-limited and iron-replete conditions. In a previous study characterizing transcriptomes during microsclerotium formation, Xiong et al. (27) reported upregulation of genes involved in carbohydrate and protein metabolic processes. In agreement, transcript levels of *cccA*, *acoA*, *lysF*, and *hemA* were highly downregulated in the Δ*VdHapX* strain. Not only are these genes involved in amino acid metabolism and respiration, they also have direct roles in vacuolar iron storage and heme biosynthesis, suggesting that VdHapX contributes to microsclerotium formation by also regulating genes related to iron acquisition and metabolism.

Ferrous iron can be rapidly oxidized to produce ferric iron and hydroxyl radicals, both of which may be toxic (28). Ferric reductases are integral membrane proteins involved in the reduction from ferric iron to ferrous iron, and the process is vital for iron uptake (29). Fungal ferric reductases not only play a role iron reduction but also are associated with sensitivity to oxidative stresses. Fungal strains lacking ferric reductases exhibited hypersensitivity to oxidative stress, i.e., in C. albicans (30), A. fumigatus (31), and V. dahliae (19). In our study, the Δ*VdHapX* strain displayed significantly increased sensitivity to H_2_O_2_. Analyses of the expression of genes associated with H_2_O_2_ detoxification also demonstrated that these key genes were downregulated in the mutant. Similarly, deletion of *HapX* in F. oxysporum led to a suppression of the expression of genes associated with oxidative stress detoxiﬁcation, such as *FOXG_12260* (peroxidase) and *FOXG_00142* (cytochrome *c* peroxidase) (6). Together, our data coupled with these previous observations clearly demonstrate that HapX is required for the iron-dependent adaptation to oxidative stress in fungi.

The critical role of iron acquisition in virulence has been shown in pathogenic fungi. HapX was shown to be important for virulence of F. oxysporum (6), A. fumigatus (5), C. albicans (10), and C. neoformans (11). As shown here, the Δ*VdHapX* strains exhibited a clear defect in virulence. Further observations showed that the mutant had delayed penetration into the cellophane membrane and onion epidermis and defective proliferation into the xylem tissues of smoke tree seedlings. However, the ties or interconnectivity between the iron regulatory system, virulence, and H_2_O_2_ detoxification are areas that remain to be explored at the genetic and biochemical levels.

## MATERIALS AND METHODS

### Fungal strains and culture conditions.

The wild-type V. dahliae strain XS11 for these experiments was isolated from a smoke tree in Fragrant Hills, Beijing, China (32). The same strain was used for preparation of the gene replacement and complementation strains in this study. The conidia of all fungal strains were stored in 30% glycerol solution at −80°C. XS11 was cultured on PDA plates, and the transformants of V. dahliae XS11 were grown on PDA plates (1 liter PDA, 200 g potato, 20 g glucose, 15 g agar) supplemented with 50 μg/ml Geneticin or 25 μg/ml hygromycin as appropriate for selection following the respective gene replacement.

To analyze the influence of iron on mutants, strains were grown on minimal medium (MM) plates (1 liter MM, 6 g NaNO_3_, 1.52 g KH_2_PO_3_, 0.52 g KCl, 0.52 g MgSO_4_·7H_2_O, 20 mM l-glutamic acid, 15 g agar) with 0.03 mM Fe_2_(SO_4_)_3_, 0.4 mM bathophenanthroline disulfonate (BPS), 5 mM FeSO_4_, or 10 mM FeSO_4_ at 25°C. For extraction of genomic DNA, fresh conidia were grown in liquid yeast extract-peptone-dextrose (YEPD) (1 liter YEPD, 3 g yeast extract, 10 g peptone, 20 g glucose) at 25°C with shaking at 200 rpm. The fresh conidia were inoculated on basal medium (BM) (1 liter BM, 10 g glucose, 0.2 g sodium nitrate, 0.52 g KCl, 0.52 g MgSO_4_·7H_2_O, 1.52 g KH_2_PO_4_, 3 μM thiamine HCl, 0.1 μM biotin, and 15 g agar) to analysis the influence of *VdHapX* on microsclerotium formation of V. dahliae.

To observe microsclerotium formation, solid BM (1 liter BM, 10 g d-glucose, 0.2 g NaNO_3_, 0.52 g KCl, 0.52 g MgSO_4_·7H_2_O, 1.52 g KH_2_PO_4_, 3 μM vitamin B_1_, 0.1 μM biotin, 15 g agar) was evenly applied to the sterile slides. Fresh conidia (0.2 μl of 1 × 10^5^ conidia/ml) of XS11, the Δ*VdHapX* strain, and the complemented strain were inoculated onto culture medium for 7 days, and the slide was placed in a sterile petri dish for 5 days at 25°C. Observation and analysis of relative ratio were performed using a microscope (Leica DM 2500) and Image J software.

For plant infection assays, all strains were grown in liquid complete medium (CM; 50 ml 20 nitrate salts, 1 ml 1,000× trace, 10 g glucose, 2 g peptone, 1 g yeast extract, 1 g Casamino Acids, 1 ml vitamin solution) at 25°C with shaking at 200 rpm for 7 days prior to use.

To test the response to peroxide, conidia were uniformly diluted with the PDA medium to 10^6^ conidia/ml and spread onto several plates. Sterilized filter paper having a diameter of 5 mm was placed in the center of the plate, and a 10-μl droplet of either a 2.5%, 5%, or 10% hydrogen peroxide solution was dropped on the filter paper pieces. Plates were held at 25°C for 7 days prior to observations of the zone of inhibition.

### Bioinformatics analysis.

The complete sequence of VdHapX was downloaded from JGI (https://genome.jgi.doe.gov/Verda1/Verda1.home.html ) by using BLASTP with the HapX gene of F. oxysporum. The reference genome of V. dahliae (23) was used for BLASTP queries of the V. dahliae genome to identify VdHapX. VdHapX orthologs in other fungi were found in NCBI and JGI. The full-length protein sequences of VdHapX homologs in different fungi were compared, and the phylogenetic tree was constructed with MEGA version 6.0 (33).

### Targeted gene knockout and mutant complementation.

The entire open reading frame region of the *VdHapX* gene was replaced with a hygromycin resistance gene cassette constructed using the split-marker method (34). The 5′ and 3′ flanking sequences of *VdHapX* were cloned with primers PL906/PL907 and PL908/PL909 and then linked with the hygromycin resistance gene cassette using primers PL906/PL938 and PL939/PL909, respectively. The hygromycin resistance cassette was sequenced by primers M13-F and M13-R. Subsequently, the 5′ sequence was connected with the hygromycin resistance gene cassette by fusion PCR with primer PL906/Hy-R, and the 3′ sequence was fused to the hygromycin resistance gene cassette by fusion PCR with the primer Yg-F/PL909. Finally, the PCR fragments were sequenced and used for polyethylene glycol (PEG)-mediated protoplast transformation as described by Wang et al. (32). The transformations were screened with 30 μg/ml hygromycin B. Mutants were detected by PCR and validated by Southern blotting. The complementation strain was selected with 50 μg/ml Geneticin by reintroducing the wild-type copy of *VdHapX*.

Genomic DNA was extracted by a cetyltrimethylammonium bromide (CTAB) method, and Southern blotting was performed to confirm the deletion of *VdHapX* by the digoxigenin (DIG) High Prime DNA labeling and detection starter kit I according to the manufacturer’s protocol (Roche, Germany). The probe fragment for the Southern blot was amplified from the V. dahliae strain XS11 genomic DNA with primers PL963 and PL907 and labeled with DIG primer. The restriction enzyme KpnI was used to digest the genomic DNA extracted from the wild-type strain and mutant strains.

### Gene expression analyses.

Total RNA was extracted from the XS11 and the Δ*VdHapX* strain using TRIzol reagent (Invitrogen) and purified with a PureLink RNA minikit (Ambion). RNA was reverse transcribed using SuperScript III reverse transcriptase (Invitrogen) and oligo(dT) to obtain cDNA used for subsequent experiments. For analyzing the expression of genes regulated by VdHapX in different strains under iron-replete or iron-limited conditions, all strains were grown in liquid MM for 3 days at 25°C and 200 rpm and transferred into liquid MM and liquid MM with 0.03 mM Fe_2_(SO_4_)_3_, respectively, for 45 min prior to RNA extraction. The mycelia were collected and frozen with liquid nitrogen immediately. Reverse transcription-qPCR was performed with the SuperReal Premix Plus (Tiangen, China) using SYBR green dye and an ABI 7500 real-time PCR system (Applied Biosystems, USA). The genes were tested independently and in triplicate. The results of RT-qPCR were analyzed using the threshold cycle (ΔΔ*C_T_*) method (35). The β-tubulin gene was used as the internal reference. The XS11 strain in liquid MM was used as a control group for all analyzes. The gene numbers and primers used in the experiments are listed in Table S1 in the supplemental material.

10.1128/mSphere.00400-18.6 TABLE S1 Primers used in this study. Download Table S1, DOCX file, 0.01 MB. Copyright © 2018 Wang et al. 2018 Wang et al. This content is distributed under the terms of the Creative Commons Attribution 4.0 International license.

For analyzing the effects of VdHapX gene knockout on reactive oxygen stress, XS11 and the Δ*VdHapX* strain were grown in liquid MM or liquid MM containing 0.015 mM Fe_2_(SO_4_)_3_ for 3 days at 25°C, respectively. Mycelia were collected and transferred into liquid MM with or without 0.015 mM Fe_2_(SO_4_)_3_ and 1 mM H_2_O_2_ after incubation for 45 min. The mycelia were collected for RNA extraction, and the methods of qPCR and data processing were the same as mentioned above. Primer sequences are listed in Table S1.

To detect the effect of deletion of the *VdHapX* gene on the ability to detoxify high concentrations of ferrous and ferric iron, XS11, the Δ*VdHapX* strain, and the Δ*VdHapX* complemented strain were first grown in liquid MM for 3 days at 25°C and 200 rpm. Mycelia were collected and transferred into liquid MM with or without 0.015 and 5 mM Fe_2_(SO_4_)_3_ for 45 min and then collected for RNA extraction. The methods of RT-qPCR and data processing were the same as described above. All primers in this assay are listed in Table S1.

### Determination of intracellular iron content.

The XS11, Δ*VdHapX*, and Δ*VdHapX*/*VdHapX* strains were inoculated on liquid MM for 3 days at 25°C and 200 rpm and were transferred to fresh MM for 45 min with or without 0.03 mM Fe^3+^. The mycelia were collected, rinsed with sterile water, and lyophilized using a vacuum freeze-dryer. The lyophilized mycelia were ground to a fine powder. Two hundred milligrams of the ground hyphae was weighed, placed in vessels of a microwave sample preparation system (Perkin Elmer, USA), and microwave digested for 2 h. The concentration of iron in the solution was determined using an optical emission spectrometer (Perkin Elmer, USA).

### Plant infection assays.

One-year-old smoke tree seedlings were used for pathogenicity assays throughout the study. Conidia from XS11, the Δ*VdHapX* strain, and the Δ*VdHapX* complemented strain were obtained and adjusted to 10^6^ conidia/ml in sterile water. Plant roots were incubated in a 10^6^-conidium/ml suspension for 10 min. Control plants were mock inoculated with distilled water. All of the plants were replanted into sterile soil, placed in a greenhouse, and observed after a period of 35 days.

To observe the ability of V. dahliae mycelium to penetrate and to form hyphopodia, cellophane membranes were overlaid on the MM plates. XS11, Δ*VdHapX*, and Δ*VdHapX* complemented strains were inoculated on cellophane for 3 days at 25°C. The cellophane was removed from the surface of the plate following incubation for 2, 4, or 6 days, and the plate was maintained for another day to observe if colonies had grown on the plates. The colonies on the cellophane were rinsed to remove the residual conidia with sterile water, allowing observations of the remaining hyphae.

The onion epidermis was soaked with alcohol and water and placed on the sterilized slides for observations of the hyphal penetration into epidermal cells. Fresh conidia (10^5^ conidia/ml) of XS11, Δ*VdHapX*, and Δ*VdHapX* complemented strains were inoculated onto the hydrophobic surface of the onion epidermis, and the sterilized slides were placed inside sterile petri dishes to them keep moist at 25°C. These slides were observed every 24 h, and the mycelium was stained with trypan blue staining solution (0.3 ml 1% trypan blue stock, 10 ml lactic acid, 10 ml phenol, 10 ml distilled water [dH_2_O]).

## References

[1] Lin H, Li L, Jia X, Ward DM, Kaplan J 2011 Genetic and biochemical analysis of high iron toxicity in yeast: iron toxicity is due to the accumulation of cytosolic iron and occurs under both aerobic and anaerobic conditions. J Biol Chem 286:3851–3862. doi:10.1074/jbc.M110.190959. 21115478 PMC3030386

[2] Halliwell B, Gutteridge JM 1984 Oxygen toxicity, oxygen radicals, transition metals and disease. Biochem J 219:1–14. doi:10.1042/bj2190001. 6326753 PMC1153442

[3] Haas H, Zadra I, Stoffler G, Angermayr K 1999 The *Aspergillus nidulans* GATA factor SREA is involved in regulation of siderophore biosynthesis and control of iron uptake. J Biol Chem 274:4613–4619. doi:10.1074/jbc.274.8.4613. 9988696

[4] Hortschansky P, Eisendle M, Al-Abdallah Q, Schmidt AD, Bergmann S, Thon M, Kniemeyer O, Abt B, Seeber B, Werner ER, Kato M, Brakhage AA, Haas H 2007 Interaction of HapX with the CCAAT-binding complex—a novel mechanism of gene regulation by iron. EMBO J 26:3157–3168. doi:10.1038/sj.emboj.7601752. 17568774 PMC1914100

[5] Schrettl M, Beckmann N, Varga J, Heinekamp T, Jacobsen ID, Jochl C, Moussa TA, Wang S, Gsaller F, Blatzer M, Werner ER, Niermann WC, Brakhage AA, Haas H 2010 HapX-mediated adaption to iron starvation is crucial for virulence of *Aspergillus fumigatus*. PLoS Pathog 6:e1001124. doi:10.1371/journal.ppat.1001124. 20941352 PMC2947994

[6] Lopez-Berges MS, Capilla J, Turra D, Schafferer L, Matthijs S, Jochl C, Cornelis P, Guarro J, Haas H, Di Pietro A 2012 HapX-mediated iron homeostasis is essential for rhizosphere competence and virulence of the soilborne pathogen *Fusarium oxysporum*. Plant Cell 24:3805–3822. doi:10.1105/tpc.112.098624. 22968717 PMC3480304

[7] Gsaller F, Hortschansky P, Beattie SR, Klammer V, Tuppatsch K, Lechner BE, Rietzschel N, Werner ER, Vogan AA, Chung D, Muhlenhoff U, Kato M, Cramer RA, Brakhage AA, Haas H 2014 The Janus transcription factor HapX controls fungal adaptation to both iron starvation and iron excess. EMBO J 33:2261–2276. doi:10.15252/embj.201489468. 25092765 PMC4232046

[8] Gsaller F, Eisendle M, Lechner BE, Schrettl M, Lindner H, Muller D, Geley S, Haas H 2012 The interplay between vacuolar and siderophore-mediated iron storage in *Aspergillus fumigatus*. Metallomics 4:1262–1270. doi:10.1039/c2mt20179h. 23151814

[9] Chen C, Pande K, French SD, Tuch BB, Noble SM 2011 An iron homeostasis regulatory circuit with reciprocal roles in *Candida albicans* commensalism and pathogenesis. Cell Host Microbe 10:118–135. doi:10.1016/j.chom.2011.07.005. 21843869 PMC3165008

[10] Hsu PC, Yang CY, Lan CY 2011 *Candida albican*s Hap43 is a repressor induced under low-iron conditions and is essential for iron-responsive transcriptional regulation and virulence. Eukaryot Cell 10:207–225. doi:10.1128/EC.00158-10. 21131439 PMC3067405

[11] Jung WH, Saikia S, Hu G, Wang J, Fung CK, D'Souza C, White R, Kronstad JW 2010 HapX positively and negatively regulates the transcriptional response to iron deprivation in *Cryptococcus neoformans*. PLoS Pathog 6:e1001209. doi:10.1371/journal.ppat.1001209. 21124817 PMC2991262

[12] Krober A, Scherlach K, Hortschansky P, Shelest E, Staib P, Kniemeyer O, Brakhage AA 2016 HapX mediates iron homeostasis in the pathogenic dermatophyte *Arthroderma benhamiae* but is dispensable for virulence. PLoS One 11:e0150701. doi:10.1371/journal.pone.0150701. 26960149 PMC4784894

[13] Wang Y, Wang Y, Tian C 2013 Quantitative detection of pathogen DNA of Verticillium wilt on smoke tree *Cotinus coggygria*. Plant Dis 97:1645–1651. doi:10.1094/PDIS-04-13-0406-RE. 30716826

[14] Klosterman SJ, Atallah ZK, Vallad GE, Subbarao KV 2009 Diversity, pathogenicity, and management of *Verticillium* species. Annu Rev Phytopathol 47:39–62. doi:10.1146/annurev-phyto-080508-081748. 19385730

[15] Bhat RG, Subbarao KV 1999 Host range specificity in *Verticillium dahliae*. Phytopathology 89:1218–1225. doi:10.1094/PHYTO.1999.89.12.1218. 18944648

[16] Pegg G, Brady B 2002 Verticillium wilts. CABI, London, United Kingdom.

[17] Singh S, Braus-Stromeyer SA, Timpner C, Tran VT, Lohaus G, Reusche M, Knufer J, Teichmann T, von Tiedemann A, Braus GH 2010 Silencing of Vlaro2 for chorismate synthase revealed that the phytopathogen *Verticillium longisporum* induces the cross-pathway control in the xylem. Appl Microbiol Biotechnol 85:1961–1976. doi:10.1007/s00253-009-2269-0. 19826808 PMC2811248

[18] Klimes A, Dobinson KF, Thomma BP, Klosterman SJ 2015 Genomics spurs rapid advances in our understanding of the biology of vascular wilt pathogens in the genus *Verticillium*. Annu Rev Phytopathol 53:181–198. doi:10.1146/annurev-phyto-080614-120224. 26047557

[19] Rehman L, Su X, Li X, Qi X, Guo H, Cheng H 2018 FreB is involved in the ferric metabolism and multiple pathogenicity-related traits of *Verticillium dahliae*. Curr Genet 64:645–659. doi:10.1007/s00294-017-0780-x. 29177887

[20] Tzima AK, Paplomatas EJ, Rauyaree P, Ospina-Giraldo MD, Kang S 2011 *VdSNF1*, the sucrose non-fermenting protein kinase gene of *Verticillium dahliae*, is required for virulence and expression of genes involved in cell wall degradation. Mol Plant Microbe Interact 24:129–142. doi:10.1094/MPMI-09-09-0217. 20839958

[21] Timpner C, Braus-Stromeyer SA, Tran VT, Braus GH 2013 The Cpc1 regulator of the cross-pathway control of amino acid biosynthesis is required for pathogenicity of the vascular pathogen *Verticillium longisporum*. Mol Plant Microbe Interact 26:1312–1324. doi:10.1094/MPMI-06-13-0181-R. 23883358

[22] Altschul SF, Gish W, Miller W, Myers EW, Lipman DJ 1990 Basic local alignment search tool. J Mol Biol 215:403–410. doi:10.1016/S0022-2836(05)80360-2. 2231712

[23] Klosterman SJ, Subbarao KV, Kang SC, Veronese P, Gold SE, Thomma BPHJ, Chen ZH, Henrissat B, Lee YH, Park J, Garcia-Pedrajas MD, Barbara DJ, Anchieta A, de Jonge R, Santhanam P, Maruthachalam K, Atallah Z, Amyotte SG, Paz Z, Inderbitzin P, Hayes RJ, Heiman DI, Young S, Zeng QD, Engels R, Galagan J, Cuomo CA, Dobinson KF, Ma LJ 2011 Comparative genomics yields insights into niche adaptation of plant vascular wilt pathogens. PLoS Pathog 7:e1002137. doi:10.1371/journal.ppat.1002137. 21829347 PMC3145793

[24] Eisendle M, Oberegger H, Zadra I, Haas H 2003 The siderophore system is essential for viability of *Aspergillus nidulans*: functional analysis of two genes encoding l-ornithine *N*^5^-monooxygenase (*sidA*) and a non-ribosomal peptide synthetase (*sidC*). Mol Microbiol 49:359–375. doi:10.1046/j.1365-2958.2003.03586.x. 12828635

[25] Mercier A, Pelletier B, Labbe S 2006 A transcription factor cascade involving Fep1 and the CCAAT-binding factor Php4 regulates gene expression in response to iron deficiency in the fission yeast *Schizosaccharomyces pombe*. Eukaryot Cell 5:1866–1881. doi:10.1128/EC.00199-06. 16963626 PMC1694796

[26] Blatzer M, Barker BM, Willger SD, Beckmann N, Blosser SJ, Cornish EJ, Mazurie A, Grahl N, Haas H, Cramer RA 2011 SREBP coordinates iron and ergosterol homeostasis to mediate triazole drug and hypoxia responses in the human fungal pathogen *Aspergillus fumigatus*. PLoS Genet 7:e1002374. doi:10.1371/journal.pgen.1002374. 22144905 PMC3228822

[27] Xiong D, Wang Y, Ma J, Klosterman SJ, Xiao S, Tian C 2014 Deep mRNA sequencing reveals stage-specific transcriptome alterations during microsclerotia development in the smoke tree vascular wilt pathogen, Verticillium dahliae. BMC Genomics 15:324. doi:10.1186/1471-2164-15-324. 24884698 PMC4035056

[28] Imlay JA 2003 Pathways of oxidative damage. Annu Rev Microbiol 57:395–418. doi:10.1146/annurev.micro.57.030502.090938. 14527285

[29] Dancis A, Klausner RD, Hinnebusch AG, Barriocanal JG 1990 Genetic evidence that ferric reductase is required for iron uptake in *Saccharomyces cerevisiae*. Mol Cell Biol 10:2294–2301. doi:10.1128/MCB.10.5.2294. 2183029 PMC360576

[30] Xu N, Qian K, Dong Y, Chen Y, Yu Q, Zhang B, Xing L, Li M 2014 Novel role of the *Candida albicans* ferric reductase gene CFL1 in iron acquisition, oxidative stress tolerance, morphogenesis and virulence. Res Microbiol 165:252–261. doi:10.1016/j.resmic.2014.03.001. 24631590

[31] Blatzer M, Binder U, Haas H 2011 The metalloreductase FreB is involved in adaptation of *Aspergillus fumigatus* to iron starvation. Fungal Genet Biol 48:1027–1033. doi:10.1016/j.fgb.2011.07.009. 21840411 PMC3188701

[32] Wang Y, Xiao S, Xiong D, Tian C 2013 Genetic transformation, infection process and qPCR quantification of *Verticillium dahliae* on smoke-tree *Cotinus coggygria*. Australas Plant Pathol 42:33–41. doi:10.1007/s13313-012-0172-0.

[33] Tamura K, Stecher G, Peterson D, Filipski A, Kumar S 2013 MEGA6: Molecular Evolutionary Genetics Analysis version 6.0. Mol Biol Evol 30:2725–2729. doi:10.1093/molbev/mst197. 24132122 PMC3840312

[34] Goswami RS 2012 Targeted gene replacement in fungi using a split-marker approach. Methods Mol Biol 835:255–269. doi:10.1007/978-1-61779-501-5_16. 22183659

[35] Livak KJ, Schmittgen TD 2001 Analysis of relative gene expression data using real-time quantitative PCR and the 2(-delta delta C(T)) method. Methods 25:402–408. doi:10.1006/meth.2001.1262. 11846609

