# Supplementary figures and images for: The Transcription Factor VdHapX Controls Iron Homeostasis and Is Crucial for Virulence in the Vascular Pathogen Verticillium dahliae
Source: mSphere. 2018 Sep 5;3(5):e00400-18. doi: 10.1128/mSphere.00400-18 (PMC6126142; doi:10.1128/mSphere.00400-18)

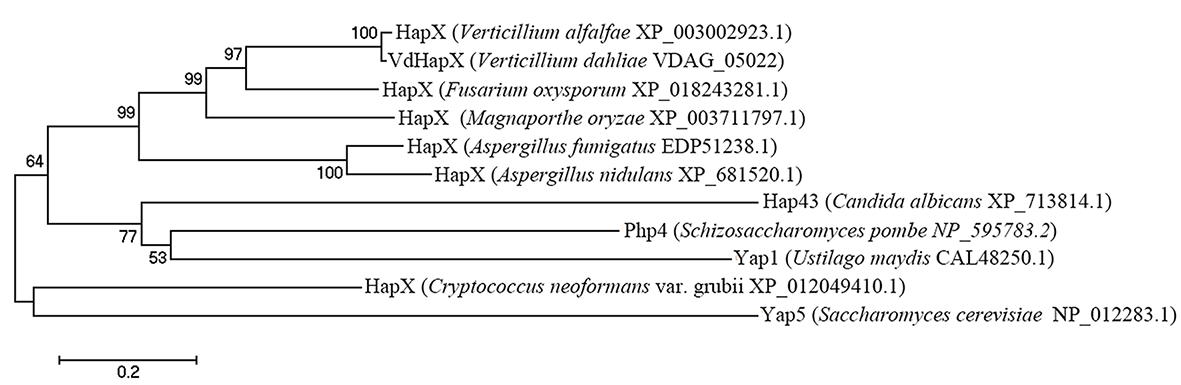

Supplement: FIG S1 [file sph004182636sf1.tif]

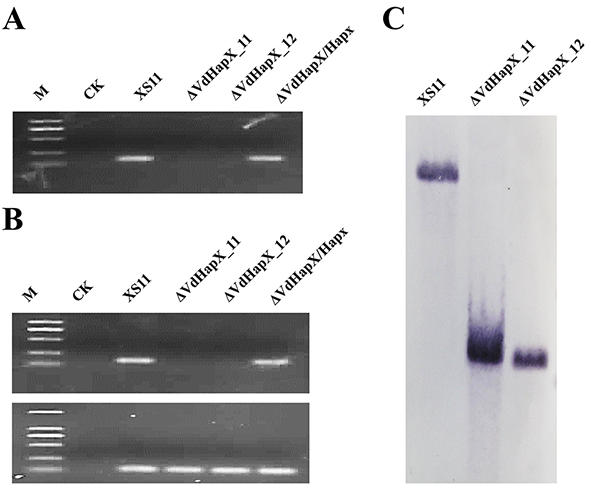

Supplement: FIG S2 [file sph004182636sf2.tif]

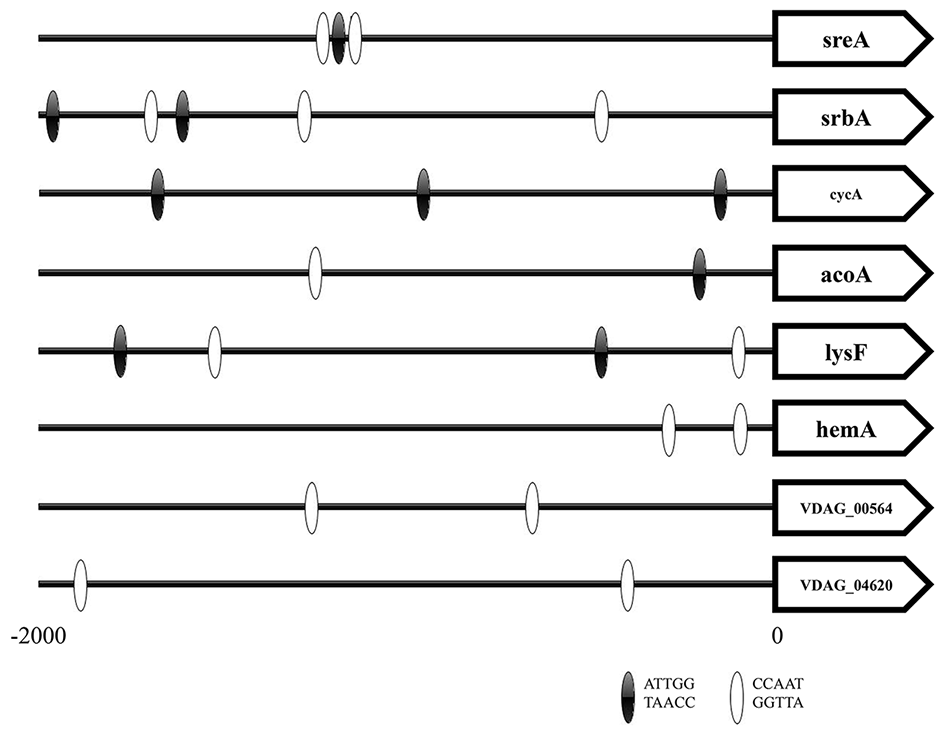

Supplement: FIG S3 [file sph004182636sf3.tif]
