# Supplementary material for: The Transcription Factor VdHapX Controls Iron Homeostasis and Is Crucial for Virulence in the Vascular Pathogen Verticillium dahliae
Source: mSphere. 2018 Sep 5;3(5):e00400-18. doi: 10.1128/mSphere.00400-18 (PMC6126142; doi:10.1128/mSphere.00400-18)
Supplement: TABLE S1 [file sph004182636st1.docx]

**Supplementary Table 1 Primers used in this study**

| Primer name | Description | Primer sequence (5'-3') |
| --- | --- | --- |
| PL906 | 5’ flanking sequence of VdHapX | TCCGATGTCGCTCAGTTT |
| PL907 |  | GTCAAGCGTTGTCCGTGT |
| PL938 |  | GTCGTGACTGGGAAAACCCTGGCGGTCAAGCGTTGTCCGTGT |
| PL908 | 3’ flanking sequence of VdHapX | GCCACCGACGACATTAGC |
| PL909 |  | GGGCAGGTTGAGTGAGATTG |
| PL939 |  | TCCTGTGTGAAATTGTTATCCGCTGCCACCGACGACATTAGC |
| PL966 | Primers for complementation of VdHapX | GTGAACCGATTATGGAACGA |
| PL967 |  | AATCACAGGCTATGGAGGC |
| PL954 | Internal screening primer of VdHapX | CGACAAGCGTCAGCGTTCC |
| PL955 |  | TTTGCAGAAGCCACAGGGAT |
| PL956 | External screening primer of VdHapX | AAACGTGCTCACCTCTTGTTAC |
| PL957 |  | GCCGAAGCGAATGTCAAA |
| HY-R | Internal primer of hygromycin- resistance cassette | GGATGCCTCCGCTCGAAGTA |
| YG-F |  | CGTTGCAAGACCTGCCTGAA |
| PL963 | Southern blot probe of VdHapX | CTTAGGCAGCGTAGTCGTG |
| PL907 |  | GTCAAGCGTTGTCCGTGT |
| PL5 | Hygromycin- resistance cassette | GGAGGTCAACACATCAATGCCT |
| PL131 |  | CCTCGGACGAGTGCTGGGGCGT |
| PL851 | qRT-PCR for VDAG_05022 | ACAAGCGTCAGCGTTCCA |
| PL852 |  | TTGCAGAAGCCACAGGGAT |
| XDG_52 | qRT-PCR for VDAG_01557 | AGCAGACGGAAGGTATGGA |
| XDG_53 |  | CTCAGGCTGGCAGTCAAA |
| XDG_48 | qRT-PCR for VDAG_00352 | GTCGTTCGCCTCACCGTCTC |
| XDG_49 |  | TCCCGCCTCTTCGCTCTGT |
| XDG_58 | qRT-PCR for VDAG_05313 | CCCGCTCCCAACAATACC |
| XDG_59 |  | TGACGCTGACGATGCTCC |
| XDG_56 | qRT-PCR for VDAG_03964 | GCTCGGCTCGTGCTTTGT |
| XDG_57 |  | AGGCCCTTGGGTATGTTCG |
| XDG_60 | qRT-PCR for VDAG_05314 | GCAAGAAGGGTTCCGAGTA |
| XDG_61 |  | CAGATTCAGCGTTGGGTAA |
| XDG_64 | qRT-PCR for VDAG_07020 | GTCACGAGCGACTTCCAATC |
| XDG_65 |  | CCACCGAAACCGACAGAG |
| XDG_54 | qRT-PCR for VDAG_02332 | GAGTTTGGTGCCGTTCCT |
| XDG_55 |  | CATCAGGCTTAATCTTGTCGT |
| XDG_66 | qRT-PCR for VDAG_08540 | AGCGTCTTCGCACTACCT |
| XDG_67 |  | CTCCAACCTTCTCCTCCC |
| XDG_62 | qRT-PCR for VDAG_06343 | AACCCGTCGCACATTATCCC |
| XDG_63 |  | CGCTTGAGGTCGAGCTTGGT |
| XDG_74 | qRT-PCR for VDAG_10085 | CGCCTCGTACAGGGAAACC |
| XDG_75 |  | CTGCTCGCAGTGCTGGAAC |
| XDG_68 | qRT-PCR for VDAG_00564 | CCGAAGACAGCCAACCTC |
| XDG_69 |  | CGCAGCTCGTGAAACAGA |
| XDG_70 | qRT-PCR for VDAG_04620 | ATCACCCAGCCCAAGTCCC |
| XDG_71 |  | CGTACCAGACGGAGGAGATGC |
| XDG_50 | qRT-PCR for VDAG_00910 | CTACTCCTACACCGATGCC |
| XDG_51 |  | CTTCTTGAGACCACCGAAA |
| VdBt-up | β-tublin of V. dahliae | AGCTCACCCAGCAGATGTTC |
| VdBt-down |  | TCGACCTCCTTCATGGCAAC |
| Yap1-F | qRT-PCR for VDAG_01588 | AACAAGCAAGCGTCTTATCC |
| Yap1-R |  | TCGCCAAAGTCATAGTCGTA |
| LY212 | qRT-PCR for VDAG_08724 | CTTCACTTCAGCAGCAACCTC |
| LY213 |  | CAGTAGGCACGGGAACGAG |
| LY214 | qRT-PCR for VDAG_03661 | TTCCAACAGCACCAGCAA |
| LY215 |  | GCAATACGGAGCCAATCA |
| LY216 | qRT-PCR for VDAG_03116 | CCAACTGGGCTCTGCTGTCT |
| LY217 |  | CGTCATCGTAGTCGTCCTTCTC |
| LY218 | qRT-PCR for VDAG_02630 | CGCTCCTCCTTACCTTGGG |
| LY219 |  | GCGTCGGTCTTGATGTTGC |
